# Perforated sarcomatoid carcinoma of the jejunum: Case report

**DOI:** 10.3892/ol.2013.1378

**Published:** 2013-06-06

**Authors:** NING HAN, QING-HUI HAN, YANG-ZHOU LIU, ZENG-CHUN LI, JIAO LI

**Affiliations:** 1 Department of Emergency and Trauma Surgery, East Hospital Affiliated to Tongji University, Shanghai 200120, P.R. China; 2 Department of Orthopaedics, Shanghai Tenth People’s Hospital Affiliated to Tongji University, Shanghai 200072, P.R. China

**Keywords:** sarcomatoid carcinoma, small intestine, perforation

## Abstract

Sarcomatoid carcinomas exhibit features that are common to epithelial and mesenchymal tumors. These carcinomas are rare, particularly in the small intestine. In the current case report, we describe a case of an intestinal sarcomatoid carcinoma in a 70-year-old Chinese female. Sarcomatoid carcinoma was confirmed based on light microscopy and immunohistochemical observations. The patient presented with symptoms of acute abdomen, which was due to an intestinal perforation caused by sarcomatoid carcinoma of the small bowel. Patients with sarcomatoid carcinoma are usually associated with a poor prognosis. However, this patient experienced a relatively favorable prognosis, which may be attributed to low positivity for Ki67 in the tumor.

## Introduction

Sarcomatoid carcinoma is a rare tumor comprised of malignant cells that possess properties of epithelial and mesenchymal carcinomas (1). In addition, sarcomatoid carcinoma has been described in various organ systems, including salivary, thyroid, breast and skin tissues, as well as the respiratory and digestive systems (1,2). Sarcomatoid carcinomas of the small intestine are rare with only a few dozen cases reported in the literature (1,3). In this case report, we present the occurrence of a sarcomatoid carcinoma in the jejunum of a 70-year-old Chinese female. Histological and immunohistochemical staining are crucial in the diagnosis of small intestinal sarcomatoid carcinoma, due to nonspecific clinical observations. To date, surgery remains the optimal therapeutic approach and previous studies have demonstrated that chemical or radial treatment do not improve the overall survival rate (1,2,4,5).

## Case report

### Clinical presentation

A 70-year-old female was hospitalized due to sudden, persistent and generalized abdominal pain that had continued for >5 h. The patient did not report nausea or vomiting. The patient’s vital signs were stable upon admission and conservative treatment, including ceftriaxone and omeprazole, did not relieve the pain. Abdominal examination revealed generalized and rebound tenderness. Digital rectal examination revealed no mass or bleeding. Evaluation of blood samples indicated an elevated white blood cell count (7.36×10^9^ cells/l) with a high percentage of neutrophils (76.9%). Abdominal X-ray revealed the possible presence of a gas shadow under the diaphragm and the computed tomography scan observations were normal. Patient consent was obtained prior to surgery.

### Surgical procedures

**Figure 1.**
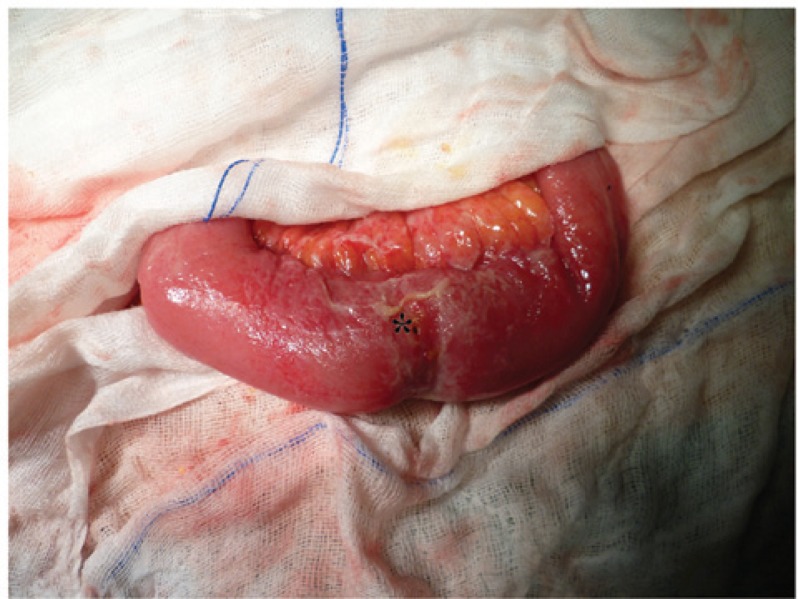
Portion of the jejunum with perforation. The intestinal wall was thickened and inflexible with a 0.1×0.1-cm perforation (indicated by an asterisk).

Immediately following the diagnosis of acute peritonitis, an exploratory laparotomy was performed that revealed a section of narrowing with an accompanying perforation (0.1×0.1 cm) in the jejunum, ∼60 cm from the Treitz ligament (Fig. 1). During the laparotomy, ∼500 ml *liquor puris* was present in the abdominal cavity. The surgeon removed a section of the jejunum (6-cm long) and performed a side-to-side small bowel anastomosis. No evidence of metastasis to the liver, spleen or stomach was observed.

### Pathological analysis

**Figure 2.**
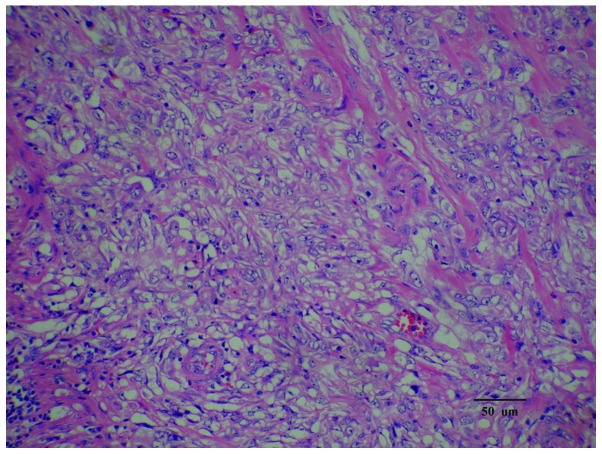
Spindle-shaped cells were present in the tumor, as well as hyper-chromatic nuclei and atypical mitotic features (staining, hematoxylin and eosin; magnification, ×200).

Pathological studies demonstrated that the tumor was characterized by spindle-shaped cells with nuclei that were heteromorphic and exhibited multiformity. In addition, karyokinesis was observed (Fig. 2). Following deparaffinization and rehydration, the sections were treated with 3% hydrogen peroxide to block endogenous peroxidase activity. Heat epitope retrieval was performed by microwave boiling the sections in 10 mmol/l citrate buffer (pH 6.0) for 12 min. Primary antibody was performed to identify vimentin, cytokeratin 8 (CK8), pan-CK, S-100, cluster of differentiation 68 (CD68), Ki67, CD34, CD117, desmin (Des), epithelial membrane antigen (EMA), leukocyte common antigen (LCA) and CD30. Samples were incubated overnight at 4°C. Immunostaining was performed with an EnVision™ system (Dako, Hamburg, Germany) on an Auto-Stainer (Dako). The antigen-antibody immunoreaction was visualized using diaminobenzedine (Dako) as a chromogen, and the slides were counterstained with Mayer’s hematoxylin. For each specimen, a negative control was obtained by omitting the primary antibody. Immunohistochemical staining revealed the following: vimentin (+++), CK8(++), pan-CK (+), S-100 (+), CD68 (+), Ki67 (5% +), CD34 (-), CD117 (-), Des(-), EMA(-), LCA (-) and CD30 (-). The diagnosis was confirmed as sarcomatoid carcinoma of the jejunum based on histological and cytological observations.

## Discussion

Sarcomatoid carcinoma is an extremely rare biphasic tumor characterized by a combination of malignant epithelial and mesenchymal cells (2). Although rare sarcomatoid carcinomas have been diagnosed in the small intestine, these tumors of the small bowel are normally diagnosed in middle-aged to older patients (mean age, 57 years), with a male to female ratio of 1.5:1 (2). Patients diagnosed with sarcomatoid carcinomas have a poor prognosis due to the high level of invasiveness of the tumor. Of these patients, ∼70% succumb to sarcomatoid carcinoma within 2 months to 3 years following the initial diagnosis (1).

Sarcomatoid carcinoma of the small bowel may be polypoid or endophytic with central ulceration. These tumors usually cause intestinal bleeding or obstruction (1,2). Reid-Nicholson *et al* previously reported a case of small bowel sarcomatoid carcinoma with perforation, in which the patient’s main complaint was melena (1). Therefore, the present case report is the first to describe sarcomatoid carcinoma in a Chinese patient that presented with acute peritonitis as the first clinical manifestation.

In this case, the diagnosis was based on pathological observations. Typically, the diagnosis is based on the observation that the sarcomatoid component is composed of epithelium-like cancer cells in a transitional zone between the sarcomatoid and carcinomatoid components. However, in this case, only poorly differentiated spindle cells were observed in the tumor. Since sarcomatoid carcinomas have only minor histological differences compared with other intestinal spindle cell tumors, the diagnosis was difficult to confirm by hematoxylin and eosin staining alone. Therefore, immunohistochemical analysis was necessary.

**Figure 3.**
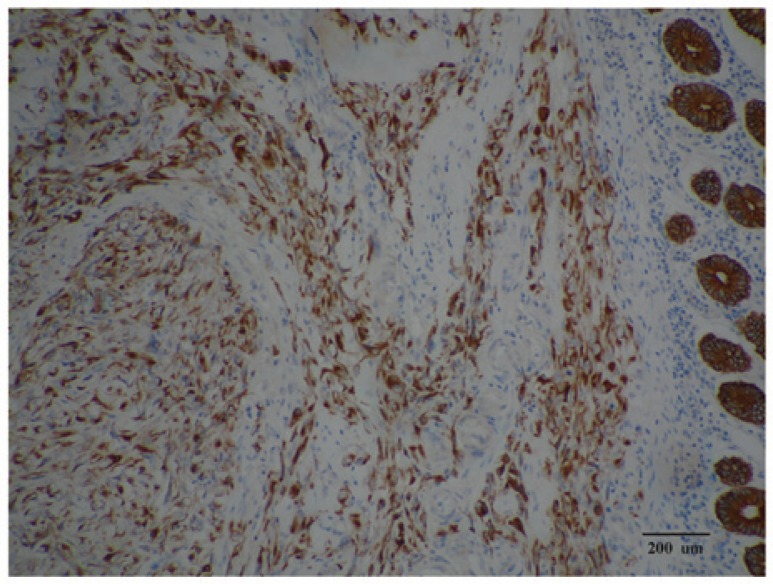
Tumor cells were markedly positive for vimentin by immunohistochemistry (staining, EnVision™ two-step method; magnification, ×200).

**Figure 4.**
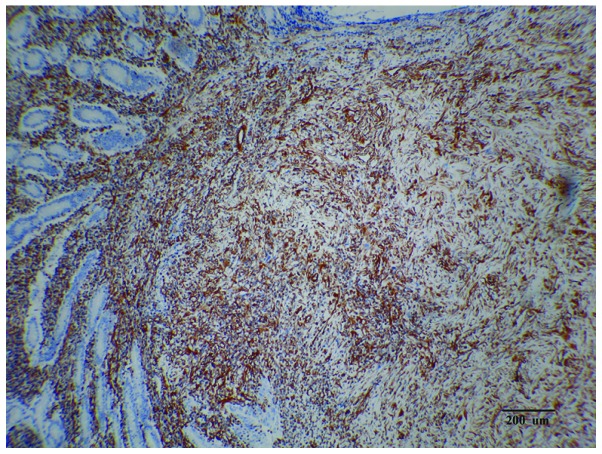
Tumor cells were markedly positive for pan-cytokeratin (CK) by immunohistochemistry (staining, EnVision™ two-step method; magnification, ×200).

Immunohistochemical staining also contributes to the diagnosis, along with routine histological examinations. In the majority of sarcomatoid carcinomas, epithelial- and mesenchymal-like components exhibit positivity for EMA. In addition, ∼90% of intestinal sarcomatoid carcinomas are positive for vimentin. Certain cases may also exhibit focal positivity for neuroendocrine and neuron-specific markers (1,2). The present case exhibited positivity for vimentin (Fig. 3), pan-CK (Fig. 4) and S100 (neuroendocrine marker), consistent with previous studies (1–3). Although the current case was negative for EMA, the diagnosis was determined based on the aforementioned observations and with the consideration of the variability of intestinal sarcomatoid carcinomas. Other intestinal spindle cell tumors typically express positivity for CD34 and CD117, as well as tumor-specific markers (2). For example, leiomyosarcoma presents with positivity for muscle-specific actin and Des (1), while these markers are negative in intestinal sarcomatoid carcinomas. Therefore, analysis of immunohistochemical staining clearly plays a decisive role in the process of differential diagnosis for sarcomatoid carcinoma.

Currently, radical excision remains the best therapy for intestinal sarcomatoid carcinomas (2,4). Previous studies have indicated that a wide excision area of the intestine around the tumor may increase the patient survival rate (2,4). In the present case, the patient exhibited serious contamination of the abdominal cavity and generalized tissue swelling prior to the surgical procedure. Therefore, only the section of the jejunum with the perforation was removed due to the poor condition of the patient. Adjuvant therapy has not been reported to improve therapeutic outcomes (1,2,4,5). In the present case, the patient refused further adjuvant therapy due to the current therapeutic progress. At the last follow-up at 7 months, the patient was living without any signs of metastasis as a result of the disease.

Ki67 is widely used to study the biological and proliferative status of various tumors. In general, higher positivity rates for Ki67 are associated with poor prognosis (6). The patient in the present case report exhibited a low positivity rate for Ki67, which may have influenced the individual’s continued favorable prognosis.

In conclusion, the possibility of perforated small bowel malignant tumor should be taken into consideration in the presence of acute abdominal pain. Furthermore, Ki67 is a potential prognosis marker for the individualized treatment of small malignant bowel tumors.
